# DVH parameters and gastrointestinal/genitourinary toxicities in moderate hypofractionated salvage radiotherapy after radical prostatectomy

**DOI:** 10.1093/jrr/rrag045

**Published:** 2026-07-07

**Authors:** Manami Otsuka, Hiroshi Taguchi, Ryusei Ashida, Hideki Minatogawa, Takuya Moriguchi, Norikata Takada, Satoru Maruyama, Toru Harabayashi, Noriaki Nishiyama

**Affiliations:** Department of Radiation Oncology, NHO Hokkaido Cancer Center, Sapporo, 4-2-3-54 Kikusui, Shiroishi-ku, Sapporo 003-0804, Japan; Department of Radiation Oncology, NHO Hokkaido Cancer Center, Sapporo, 4-2-3-54 Kikusui, Shiroishi-ku, Sapporo 003-0804, Japan; Department of Radiation Oncology, NHO Hokkaido Cancer Center, Sapporo, 4-2-3-54 Kikusui, Shiroishi-ku, Sapporo 003-0804, Japan; Department of Radiation Oncology, NHO Hokkaido Cancer Center, Sapporo, 4-2-3-54 Kikusui, Shiroishi-ku, Sapporo 003-0804, Japan; Department of Urology, NHO Hokkaido Cancer Center, Sapporo, 4-2-3-54 Kikusui, Shiroishi-ku, Sapporo 003-0804, Japan; Department of Urology, NHO Hokkaido Cancer Center, Sapporo, 4-2-3-54 Kikusui, Shiroishi-ku, Sapporo 003-0804, Japan; Department of Urology, NHO Hokkaido Cancer Center, Sapporo, 4-2-3-54 Kikusui, Shiroishi-ku, Sapporo 003-0804, Japan; Department of Urology, NHO Hokkaido Cancer Center, Sapporo, 4-2-3-54 Kikusui, Shiroishi-ku, Sapporo 003-0804, Japan; Department of Radiation Oncology, NHO Hokkaido Cancer Center, Sapporo, 4-2-3-54 Kikusui, Shiroishi-ku, Sapporo 003-0804, Japan

**Keywords:** prostate cancer, salvage radiotherapy, hypofractionation, dose–volume histogram, anal canal, rectal bleeding

## Abstract

This study aimed to evaluate the relationships between dose–volume histogram (DVH) parameters and gastrointestinal (GI) and genitourinary toxicity in patients who underwent moderate hypofractionated salvage radiotherapy after radical prostatectomy. We retrospectively reviewed patients who received moderate hypofractionated salvage radiotherapy (65 Gy in 26 fractions, four fractions per week) between 2013 and 2022. DVH parameters of the rectum, anal canal, whole bladder, bladder neck, and bladder trigone were assessed in relation to toxicity. Optimal cut-off values were determined using receiver operating characteristic curve analysis. Ninety-two patients were included, with a median follow-up of 5.2 years. Grade ≥ 2 acute rectal bleeding, acute anal pain, late rectal bleeding, and grade ≥ 1 late hematuria occurred in 13%, 24%, 14%, and 33% of patients, respectively. Acute GI toxicity was associated with low-to-moderate doses to the anal canal, whereas late rectal bleeding was associated with moderate-to-high doses to the rectum. In multivariable analysis, anal canal V20Gy and hypertension were independent predictors of acute rectal bleeding, and rectum V60Gy and acute rectal bleeding were independently associated with late rectal bleeding. These findings may contribute to establishing DVH thresholds that facilitate dose optimization in moderate hypofractionated salvage radiotherapy after radical prostatectomy.

## INTRODUCTION

Biochemical recurrence occurs in approximately 30% of patients after radical prostatectomy [1], and salvage radiotherapy has been shown to improve prostate cancer-specific survival [2]. However, the optimal dose constraints for organs at risk (OARs) in the postoperative setting remain undefined. Although several studies have evaluated dose–volume histogram (DVH) parameters in relation to gastrointestinal (GI) toxicity and genitourinary (GU) toxicities, the available evidence remains limited [3, 4], particularly for hypofractionated salvage therapy.

Moreover, moderate hypofractionated salvage radiotherapy has been associated with a risk of severe late adverse events [5, 6]. Ranta et al. [5] reported that late toxicity is highly time-dependent. Although only two cases of severe toxicity were observed within 2 years after treatment, the cumulative incidence gradually increased, ultimately reaching 34% at 15 years for grade 3–5 late adverse events, with a median time to onset of 106 months.

Since 2008, our institution has used a regimen of 65 Gy in 26 fractions, modified from 60 Gy in 24 fractions, with the aim of potentially improving local control. Compared with standard salvage regimens (64–66 Gy in 32–33 fractions), this fractionation schedule should be considered not merely moderate hypofractionation, but a dose-escalated regimen. In terms of radiobiology, the equivalent dose in 2-Gy fractions (EQD2; α/β = 3.0 Gy) is 71.5 Gy, and the biologically effective dose (BED3) is 119.2 Gy, both of which are higher than those of standard fractionation. Therefore, this regimen may increase the risk of late adverse events. Notably, the phase III SAKK 09/10 trial demonstrated that dose escalation in salvage radiotherapy (70 Gy vs. 64 Gy) did not improve biochemical control but significantly increased late GI toxicity [7].

In definitive radiotherapy for prostate cancer, higher doses to specific bladder subregions—such as the bladder neck and trigone—have been reported to correlate with GU toxicity [8, 9], whereas doses to the anal canal have been associated with acute GI toxicity [10]. Similar associations between bladder subregion dose and GU toxicity have also been reported in other pelvic malignancies [11, 12]. These findings suggest that evaluating not only whole-organ doses but also regional dose distributions within the bladder and rectum may improve the prediction of treatment-related toxicity. However, these region-based dosimetric analyses have not been sufficiently investigated in the postoperative setting. The goal of this study was to comprehensively assess the associations between DVH parameters of the rectum, anal canal, whole bladder, bladder neck, and bladder trigone and the incidence of GI and GU toxicity in patients receiving moderate hypofractionated radiotherapy (65 Gy in 26 fractions, four fractions per week). Our goal was to generate clinically relevant dosimetric data that could facilitate the formulation of dose-constraint recommendations for moderate hypofractionated salvage radiotherapy after radical prostatectomy.

## MATERIALS AND METHODS

### Patients

In this study, patients who underwent salvage radiotherapy for biochemical recurrence after radical prostatectomy at our institution between January 2013 and December 2022 were included. Patients with a follow-up period of < 6 months or those with macroscopic recurrence identified on imaging (CT or MRI) were excluded. This study was approved by the institutional ethics review board (approval No. 06–52; date of approval: February 3, 2025), and all procedures were performed in compliance with the Declaration of Helsinki, relevant laws, and institutional guidelines while ensuring full protection of individual privacy. Given the retrospective design and the use of anonymized data, the requirement for written informed consent was waived by the institutional ethics review board.

### Radiotherapy

Computed tomography (CT) simulation was performed with a slice thickness of 2.5 mm. Patients were instructed to drink 200 ml of water approximately 30 minutes before CT simulation and before each treatment session. Since the introduction of intensity-modulated radiation therapy (IMRT), patients have generally been instructed to take laxatives beginning 1 week prior to CT simulation.

All patients received 65 Gy in 26 fractions (2.5 Gy per fraction), delivered four fractions per week. The clinical target volume (CTV) was defined as the prostate bed alone, excluding the pelvic lymph node regions. The CTV was delineated in accordance with the European Organisation for Research and Treatment of Cancer (EORTC) consensus guidelines [13] and included the vesicourethral anastomosis (VUA) and bladder neck. The lateral border was defined by the obturator internus muscle, the anterior border was defined by the posterior bladder wall, and the posterior border was defined by the anterior rectal wall. For patients with seminal vesicle invasion (pT3b), the seminal vesicle bed was also included in the CTV. The planning target volume (PTV) was generated by adding a 7–10 mm margin to the CTV.

Before September 2021, three-dimensional conformal radiation therapy (3D-CRT) was employed. In terms of 3D-CRT planning, the multileaf collimator leaf margin was set to 5 mm from the PTV. 3D-CRT was delivered using either a 7-field technique or a 7-direction, 11-field field-in-field (FIF) technique. The beam arrangement consisted of gantry angles of 0°, 55°, 100°, 135°, 225°, 260°, and 305°. When further reduction of the rectal dose was deemed necessary, the 7-direction, 11-field FIF technique was selected, and additional FIF beams were applied at 55°, 135°, 225°, and 305°. Radiotherapy was delivered using multiple linear accelerators, including the Clinac 600CD, Clinac 2100C, Clinac IX, and TrueBeam STx (Varian Medical Systems, Palo Alto, CA, USA). No explicit numeric dose constraints were applied to OARs in 3D-CRT planning. Image guidance was performed using skin marks and bony anatomy matching.

With respect to IMRT, daily image guidance was performed using cone-beam CT (CBCT). Patient setup was verified using bony anatomy, followed by soft tissue matching based on soft tissue structures in the prostatic fossa, such as the VUA and the posterior bladder wall. The dose constraints for IMRT were based on institutional protocols for definitive prostate radiotherapy, developed with reference to the Kyoto University protocol [14], and were adapted for the salvage setting.

The dose constraints for IMRT were as follows:

• PTV: D50 = 65 Gy, D95% > 58.5 Gy, D2% < 68.3 Gy.

• Rectal wall: V40Gy < 60%, V55Gy < 40%, V60Gy < 20%, D5cc < 63 Gy, D2% < 60 Gy.

• Bladder wall: V40Gy < 60%, D2% < 65 Gy.

• Penile bulb: Dmean <42 Gy.

### OAR delineation and DVH analysis

The rectal wall and bladder wall were contoured as 4-mm-thick structures from the outer contour for treatment planning. For DVH analysis in this study, the rectum, anal canal, whole bladder, bladder neck, and bladder trigone were retrospectively delineated on planning CT imaging in accordance with previously published guidelines [15, 16], without the use of MRI fusion. The rectum was contoured from the superior border of the anal canal to the rectosigmoid junction. The anal canal was delineated from the anal verge extending 3 cm cranially. The entire bladder volume was contoured. The bladder neck was contoured as a 4-mm-thick bladder wall extending 1 cm cranially from the VUA, defined as the level at which urine was observed. The bladder trigone was defined as the posterior 4-mm-thick bladder wall bounded by the bilateral ureters and the VUA. An example of contouring is shown in Supplementary Fig. S1.

All OARs were contoured by a single radiation oncologist and subsequently reviewed and modified by another radiation oncologist. DVH data were extracted from the Eclipse treatment planning system (Varian Medical Systems, Palo Alto, CA, USA). The following DVH parameters of each organ were calculated: Dmax, D0.1cc, D2cc, Dmean, V10Gy–V65Gy (%), and organ volume (cc).

### Follow-up and toxicity evaluation

Patients were examined weekly during radiotherapy and then every 3–6 months after treatment was completed. Adverse events were retrospectively collected from medical records and graded according to the Common Terminology Criteria for Adverse Events version 5.0. Toxicities that occurred within 3 months after the completion of radiotherapy were defined as acute, and those that occurred thereafter were defined as late. The evaluated endpoints included rectal bleeding, anal pain, and hematuria.

### Statistical analysis

Univariate logistic regression analyses were performed to evaluate the associations between DVH parameters and each toxicity. Odds ratios (ORs) for DVH parameters were calculated per one-unit increase (1 Gy, 1%, or 1 cc). DVH parameters that were statistically significant in univariate analysis were further evaluated using receiver operating characteristic (ROC) curve analysis to determine optimal cut-off values. Variables with *P* < 0.1 in the univariate analysis were considered candidates for multivariate analysis. However, because of the limited number of events, the number of variables included in the multivariate model was limited to two to decrease the risk of overfitting, with one clinically relevant variable and one DVH parameter selected. The probability of late toxicity was estimated using the Kaplan–Meier method, and multivariable analyses were performed using Cox proportional hazards models. Biochemical failure-free survival (BFFS), overall survival (OS), and metastasis-free survival (MFS) were estimated using the Kaplan–Meier method. Biochemical failure after salvage radiotherapy was defined as a prostate-specific antigen (PSA) level ≥ 0.4 ng/ml. A *P* value <0.05 was considered to indicate statistical significance. All statistical analyses were performed using JMP version 18 (SAS Institute, Cary, NC, USA).

## RESULTS

A total of 92 patients were included in the analysis. The median age was 69 years (interquartile range [IQR], 67–72), and the median follow-up duration was 5.2 years (IQR, 3.8–7.3). The median PSA concentration before radiotherapy was 0.35 ng/ml (IQR, 0.30–0.45 ng/ml). 3D-CRT was used in 80 patients (87%), and IMRT was used in 12 patients (13%). Seven patients (7.6%) received concurrent hormone therapy (Table 1). The 5-year BFFS, OS, and MFS rates were 61.8%, 97.6%, and 88.9%, respectively (Supplementary Fig. S2).

**Table 1:** Patient and treatment characteristics.

Variable		Median (IQR) or n (%)	
Age (years)		69 (67–72)	
Pre-RT PSA (ng/ml)		0.35 (0.30–0.45)	
Time from surgery to RT (months)		43.6 (24.7–75.2)	
Follow-up period (years)		5.2 (3.8–7.3)	
Gleason score			
6		9 (9.8%)	
7		70 (76.1%)	
≥ 8		12 (13.0%)	
Not available		1 (1.1%)	
T stage			
pT2		48 (52.2%)	
pT3a		27 (29.3%)	
pT3b		14 (15.2%)	
pT4		1 (1.1%)	
pTx		2 (2.2%)	
Resection margins			
Positive		43 (46.7%)	
Negative		47 (51.1%)	
Not available		2 (2.2%)	
RT technique			
3D-CRT		80 (87.0%)	
IMRT		12 (13.0%)	
Concurrent ADT		7 (7.6%)	
Comorbidities			
Diabetes		16 (17.4%)	
Hypertension		41 (44.6%)	
Anticoagulant use		9 (9.8%)	
Hemorrhoid		24 (26.1%)	

**Abbreviations:** IQR = interquartile range; RT = radiotherapy; PSA = prostate-specific antigen; 3D-CRT = three-dimensional conformal radiotherapy; IMRT = intensity-modulated radiation therapy; ADT = androgen deprivation therapy.

**Fig. 1:**
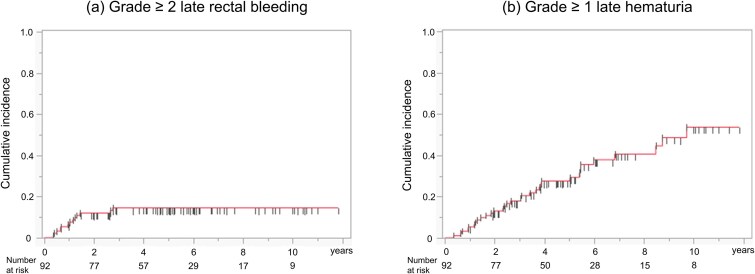
Kaplan–Meier curves for cumulative incidence of late toxicities. (a) The cumulative incidence of grade ≥ 2 late rectal bleeding was 14.6% at 5 years and remained unchanged at 10 years. (b) The cumulative incidence of grade ≥ 1 late hematuria was 27.6% and 53.8% at 5 years and 10 years, respectively.

Adverse events are summarized in Supplementary Table S1. Grade 1 and 2 acute rectal bleeding occurred in 6 (6.5%) and 12 (13.0%) patients, respectively. Grade 1, 2, and 3 late rectal bleeding occurred in 22 (23.9%), 5 (5.4%), and 8 (8.7%) patients, respectively. Acute anal pain occurred in 14 (15.2%) patients with grade 1 toxicity and 22 (23.9%) patients with grade 2 toxicity. Late hematuria occurred in 28 (30.4%) patients with grade 1 toxicity, 2 (2.2%) patients with grade 2 toxicity, and 3 (3.3%) patients with grade 3 toxicity. No grade ≥ 4 toxicity was observed. The cumulative incidence of grade ≥ 2 late rectal bleeding was 14.6% at 5 years and remained unchanged at 10 years. The cumulative incidence of grade ≥ 1 late hematuria increased over time, reaching 27.6% at 5 years and 53.8% at 10 years (Fig. 1).

### Associations between DVH parameters and toxicity

#### Grade ≥ 2 acute rectal bleeding

**Fig. 2:**
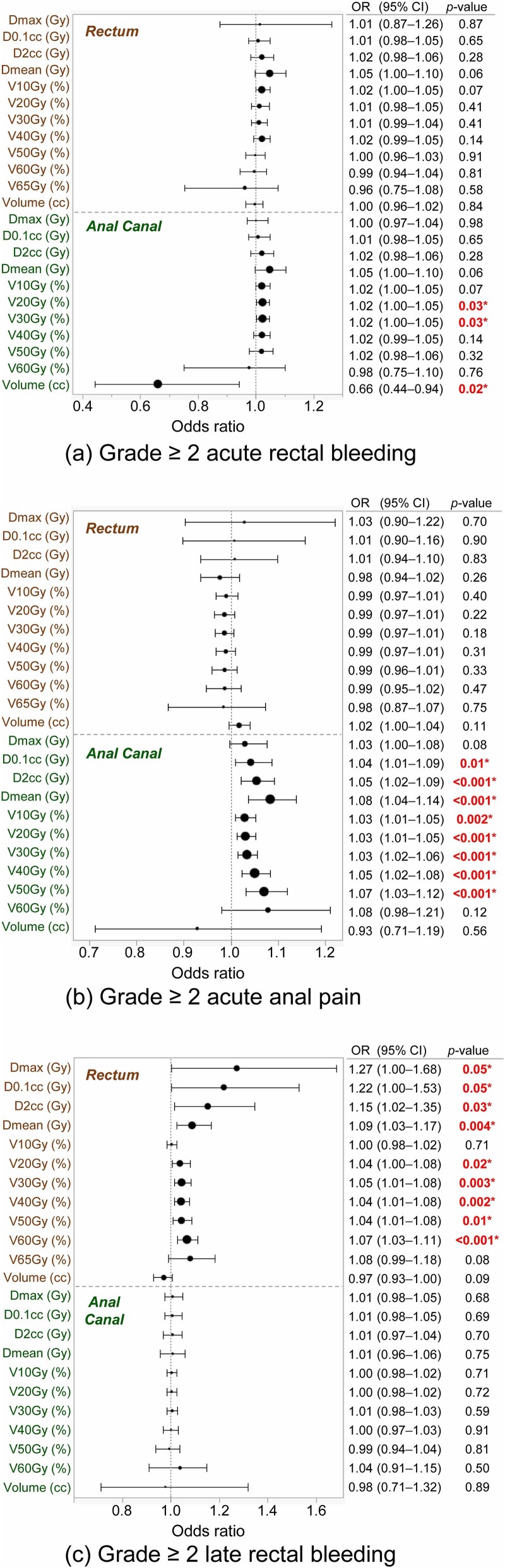
Univariate logistic regression analyses of DVH parameters associated with gastrointestinal toxicity: (a) grade ≥ 2 acute rectal bleeding, (b) grade ≥ 2 acute anal pain, (c) grade ≥ 2 late rectal bleeding**.** Because the distributions of anal canal V65Gy were highly skewed, the corresponding odds ratio (OR) estimates were unstable and could not be calculated. *P* < 0.05 is shown in bold with an asterisk (^*^). **Abbreviations:** OR = odds ratio, CI = confidence interval.

Univariate analysis of the DVH parameters revealed a significant association with anal canal V20Gy, V30Gy, and anal canal volume (Fig. 2a). ROC curve analysis identified anal canal V20Gy ≥ 49.6%, anal canal V30Gy ≥ 13.6%, and anal canal volume ≤ 6.2 cc as the optimal cut-off (Table 2). Multivariate analysis revealed that anal canal V20Gy (OR 1.04 per 1%, 95% CI 1.01–1.08; *P* < 0.001) and hypertension (OR 16.83, 95% CI 3.30–144.85; *P* = 0.003) were independent risk factors (Table 3a).

**Table 2:** Cutoff values of DVH parameters and incidence of toxicities.

DVH Parameter	Cutoff	AUC (95% CI)	Sensitivity	Specificity
Grade ≥ 2 acute rectal bleeding				
Anal canal V20Gy	≥ 49.6%	0.67 (0.48–0.85)	0.67	0.70
Anal canal V30Gy	≥ 13.6%	0.66 (0.47–0.84)	0.83	0.51
Anal canal volume	≤ 6.2 cc	0.70 (0.52–0.87)	0.92	0.50
Grade ≥ 2 acute anal pain				
Anal Canal D0.1cc	≥ 54.0 Gy	0.71 (0.59–0.83)	0.68	0.70
Anal Canal D2cc	≥ 25.5 Gy	0.73 (0.60–0.85)	0.73	0.73
Anal Canal Dmean	≥ 20.2 Gy	0.74 (0.62–0.86)	0.68	0.73
Anal Canal V10Gy	≥ 68.8%	0.73 (0.60–0.85)	0.68	0.73
Anal Canal V20Gy	≥ 60.4%	0.72 (0.60–0.84)	0.55	0.84
Anal Canal V30Gy	≥ 20.7%	0.73 (0.61–0.85)	0.68	0.70
Anal Canal V40Gy	≥ 32.9%	0.73 (0.61–0.85)	0.45	0.94
Anal Canal V50Gy	≥ 10.1%	0.72 (0.60–0.84)	0.45	0.86
Grade ≥ 2 late rectal bleeding				
Rectum Dmax	≥ 64.9 Gy	0.73 (0.56–0.89)	0.69	0.78
Rectum D0.1cc	≥ 64.6 Gy	0.73 (0.56–0.89)	0.69	0.81
Rectum D2cc	≥ 63.8 Gy	0.73 (0.56–0.89)	0.69	0.78
Rectum Dmean	≥ 45.6 Gy	0.73 (0.56–0.89)	0.54	0.87
Rectum V20Gy	≥ 80.5%	0.67 (0.50–0.85)	0.69	0.83
Rectum V30Gy	≥ 78.9%	0.74 (0.57–0.90)	0.62	0.81
Rectum V40Gy	≥ 55.0%	0.74 (0.57–0.90)	0.69	0.76
Rectum V50Gy	≥ 49.7%	0.70 (0.52–0.87)	0.54	0.89
Rectum V60Gy	≥ 19.6%	0.75 (0.57–0.90)	0.69	0.84
Grade ≥ 1 late hematuria				
Bladder volume	≥ 125.2 cc	0.61 (0.49–0.73)	0.45	0.79

**Abbreviations:** AUC = area under the curve; CI = confidence interval.

**Table 3:** Univariate and multivariate analyses of toxicities using logistic regression for acute toxicity and cox proportional hazards models for late toxicity.

(a) Grade ≥ 2 acute rectal bleeding						
	Univariate analysis			Multivariate analysis		
Factor	*P-*value	OR	(95% CI)	*P-*value	OR	(95% CI)
Anticoagulant use	0.85	0.82	(0.04–5.13)			
HT	0.003^*^	7.90	(1.92–53.69)	0.003^*^	16.83	(3.30–144.85)
DM	0.47	1.72	(0.35–6.71)			
Age	0.96	1.00	(0.90–1.12)			
Hemorrhoid	0.21	2.29	(0.62–8.04)			
ADT	0.26	3.00	(0.39–16.14)			
IMRT vs. 3D-CRT	0.05^*^	4.50	(1.02–18.13)			
Pathological stage ≥ T3b	0.37	0.42	(0.02–2.42)			
Anal canal V20Gy (%)	0.03^*^	1.02	(1.00–1.05)	<0.001^*^	1.04	(1.01–1.08)
(b) Grade ≥ 2 acute anal pain						
	Univariate analysis					
Factor	*P-*value	OR	(95% CI)			
Anticoagulant use	0.31	0.37	(0.02–2.19)			
HT	0.92	1.05	(0.39–2.75)			
DM	0.91	1.07	(0.27–3.53)			
Age	0.46	1.03	(0.95–1.13)			
Hemorrhoid	0.22	1.93	(0.67–5.38)			
ADT	0.77	1.30	(0.18–6.56)			
IMRT vs. 3D-CRT	0.43	1.72	(0.42–6.16)			
Pathological stage ≥ T3b	0.25	0.42	(0.06–1.71)			
Anal canal V40Gy (%)	<0.001^*^	1.05	(1.02–1.08)			
(c) Grade ≥ 2 late rectal bleeding						
	Univariate analysis			Multivariate analysis		
Factor	*P-*value	HR	(95% CI)	*P-*value	HR	(95% CI)
Anticoagulant use	0.009^*^	4.82	(1.48–15.67)			
HT	0.02^*^	4.75	(1.31–17.28)			
DM	0.93	0.93	(0.21–4.23)			
Age	0.29	1.05	(0.96–1.16)			
Hemorrhoid	0.38	0.50	(0.11–2.28)			
ADT	0.95	0.93	(0.12–7.18)			
IMRT vs. 3D-CRT	0.60	0.58	(0.08–4.47)			
Pathological stage ≥ T3b	0.50	1.56	(0.43–5.68)			
Grade ≥ 2 acute bleeding	0.003^*^	5.28	(1.72–16.17)	0.002^*^	5.92	(1.91–18.31)
Rectum V60Gy (%)	0.001^*^	1.06	(1.02–1.09)	0.001^*^	1.06	(1.03–1.10)
(d) Grade ≥ 1 late hematuria						
	Univariate analysis			Multivariate analysis		
Factor	*P-*value	HR	(95% CI)	*P-*value	HR	(95% CI)
Anticoagulant use	0.41	1.50	(0.58–3.90)			
HT	0.68	1.16	(0.58–2.32)			
DM	0.52	1.32	(0.57–3.05)			
Age	0.42	0.98	(0.93–1.04)			
ADT	0.36	1.75	(0.53–5.82)			
IMRT vs. 3D-CRT	0.02^*^	3.46	(1.23–9.71)	0.02^*^	3.41	(1.21–9.64)
Pathological stage ≥ T3b	0.27	0.51	(0.16–1.69)			
Bladder volume	0.03^*^	1.01	(1.00–1.01)	0.04^*^	1.01	(1.00–1.01)

*P* < 0.05 is shown in bold with an asterisk (^*^).

**Abbreviations:** OR = odds ratio; CI = confidence interval; Anticoagulant use = anticoagulant and antiplatelet agent use; HT = hypertension; DM = diabetes mellitus; ADT = androgen deprivation therapy; IMRT = intensity-modulated radiation therapy; 3D-CRT = three-dimensional conformal radiotherapy; HR = hazard ratio.

#### Grade ≥ 2 acute anal pain

Acute anal pain was significantly associated with several low-to-moderate dose anal canal DVH parameters, including D0.1cc, D2cc, Dmean, and V10Gy–V50Gy (Fig. 2b). ROC curve analysis revealed several cut-off values; representative examples included anal canal Dmean ≥20.2Gy, V20Gy ≥ 60.4%, and V40Gy ≥ 32.9% (Table 2). No clinical factors were significantly associated with acute anal pain; therefore, multivariate analysis was not performed (Table 3b).

#### Grade ≥ 2 late rectal bleeding

Late rectal bleeding was significantly associated with several rectal DVH parameters, including Dmax, D0.1cc, D2cc, Dmean, and V20Gy–V60Gy (Fig. 2c). ROC curve analysis revealed several cut-off values; representative examples included rectum D2cc ≥ 63.8 Gy, V40Gy ≥ 55.0%, and V60Gy ≥ 19.6% (Table 2). Multivariable analysis showed that rectum V60Gy (HR 1.06 per 1%, 95% CI 1.03–1.10; *P* = 0.001) and grade ≥ 2 acute bleeding (HR 5.92, 95% CI 1.91–18.31; *P* = 0.002) were independent risk factors (Table 3c).

#### Grade ≥ 1 late hematuria

Although no significant association was observed between DVH parameters and late hematuria in the univariate analysis, a marginal trend was noted for whole bladder volume (Supplementary Fig. S3). ROC analysis revealed a bladder volume ≥ 125.2 cc as the optimal cut-off value (Table 2). Multivariate analysis revealed that IMRT use (HR 3.41, 95% CI 1.21–9.64; *P* = 0.02) and larger bladder volume (HR 1.01 per 1 cc, 95% CI 1.00–1.01; *P* = 0.04) were independent risk factors (Table 3d).

## DISCUSSION

To our knowledge, this is the first study to systematically evaluate the relationships between GI/GU toxicity and DVH parameters by anatomical subregion in patients treated with moderate hypofractionated salvage radiotherapy after radical prostatectomy. Previous studies of postoperative prostate radiotherapy have focused mainly on assessing whole-organ doses to the rectum or bladder. In contrast, we analyzed the anal canal, bladder neck, and bladder trigone in detail. Our findings revealed that acute GI toxicity was significantly associated with low-to-moderate doses to the anal canal, whereas late rectal bleeding was associated with moderate-to-high doses to the rectum.

The multivariate analysis revealed that grade ≥ 2 acute rectal bleeding was independently associated with hypertension and anal canal V20Gy. Grade ≥ 2 anal pain was associated with anal canal D0.1cc, D2cc, Dmean, and V10Gy–V50Gy. Previous studies of definitive prostate radiotherapy, including a limited number of postoperative cases, have similarly reported associations between anal canal V20Gy and acute anal symptoms [10]. Comparable associations involving anal canal V10Gy–V40Gy have also been observed in other pelvic tumors [17, 18]. Hypertension may contribute to rectal bleeding by promoting vascular endothelial injury and reducing the tissue repair capacity [19]. In the randomized trial NRG-GU003 [20], which compared moderate hypofractionated radiotherapy (62.5 Gy in 25 fractions) with conventional fractionation (66.6 Gy in 37 fractions), the EPIC bowel quality-of-life (QOL) score significantly decreased for the hypofractionated arm at the end of radiotherapy. These findings suggest that, in terms of moderate hypofractionated salvage radiotherapy, contouring the anal canal as an OAR and applying dose constraints such as V20Gy < 50% and V40Gy < 33% may reduce acute GI toxicity and better preserve patient QOL.

The multivariate analysis revealed that grade ≥ 2 late rectal bleeding was independently associated with rectum V60Gy and acute rectal bleeding. In conventionally fractionated postoperative radiotherapy (64 Gy in 32 fractions), rectum V50Gy ≥ 33% and V60Gy ≥ 13% have been reported to be associated with grade ≥ 1 late rectal bleeding [3]. Another postoperative radiotherapy study (70 Gy in 35 fractions) similarly revealed associations between rectum V35Gy, V63Gy, and rectal bleeding [4], which is consistent with our findings on dose–response relationships. Furthermore, previous studies [21, 22] have reported that acute rectal bleeding is a significant predictor of late rectal bleeding, suggesting that acute inflammation may promote tissue fibrosis and vascular damage, ultimately leading to late bleeding. Although no significant association was observed between the anal canal dose and late rectal bleeding in the present study, minimizing the anal canal dose to prevent acute bleeding may help reduce the risk of late toxicity. The cut-off values obtained from the ROC curve analysis—rectum V60Gy < 20%, V40Gy < 55%, and D2cc < 64 Gy—may be considered potential reference values for dose optimization in terms of moderate hypofractionated salvage radiotherapy delivered in 26 fractions.

The multivariate analysis revealed that grade ≥ 1 late hematuria was independently associated with IMRT use and larger bladder volume. Although a previous study reported bladder V50Gy ≥ 43% and V40Gy ≥ 50% as risk factors for hematuria after postoperative radiotherapy [4], these findings were not observed in the present study. The observed associations between IMRT use and larger bladder volume and an increased risk of hematuria should be interpreted with caution. First, a large number of patients in this cohort were treated without daily CBCT-guided IGRT, and bladder volume at treatment delivery may not have been consistent with that during planning. Second, the number of patients treated with IMRT was small (*n* = 12), which may have increased the risk of statistical instability. To address this shortcoming, a supplementary analysis limited to patients treated with 3D-CRT (*n* = 80) was performed. The associations between DVH parameters and toxicities remained consistent with those observed in the overall cohort (Supplementary Table S2). Furthermore, grade ≥ 1 hematuria was selected as the endpoint to ensure sufficient statistical power; however, because this definition includes asymptomatic and nonspecific findings, its clinical relevance is limited. Accordingly, the results of this analysis should be interpreted as exploratory and hypothesis-generating.

In this study, the cumulative incidence of grade ≥ 2 late rectal bleeding was 14.6% at 5 years and remained unchanged thereafter, suggesting that most events occurred within the first few years after treatment. In contrast, the cumulative incidence of grade ≥ 1 late hematuria gradually increased, reaching 53.8% at 10 years. These findings are consistent with previous reports [5], which indicate that late GU toxicity is highly time-dependent. Accordingly, the incidence of adverse events in our study was relatively higher than that in previous reports [3]. Therefore, these findings may be relevant in settings where similar dose-escalated hypofractionated regimens are employed.

In this study, a 4-fractions-per-week irradiation schedule was adopted. At our institution, this schedule was routinely used for external beam radiation therapy for operational reasons, including workflow efficiency. From a radiobiological perspective, prolongation of the overall treatment time may reduce acute toxicity by promoting the repair of sublethal damage in normal tissues; however, it is unlikely to reduce the risk of late toxicity. Consistent with this concept, the phase II randomized PATROIT trial of stereotactic radiotherapy for prostate cancer, which compared once-weekly irradiation with 2–3 fractions per week, demonstrated that once-weekly irradiation significantly improved acute GI and GU QOL with no significant difference in late toxicity [23].

Several limitations should be acknowledged. First, this was a single-institution retrospective study with a limited sample size. Second, the bladder neck was contoured without MRI fusion. Because CT has been reported to identify the VUA more cranially than MRI [24], the dosimetric assessment of the bladder neck may have been affected. Third, daily CBCT-guided IGRT was not performed in many cases, which may have affected the reproducibility of bladder volume. Fourth, toxicity data were retrospectively collected from medical records, and patient-reported outcomes were not systematically obtained.

This study reported that, in moderate hypofractionated salvage radiotherapy after radical prostatectomy, low-to-moderate anal canal doses were associated with acute rectal bleeding and anal pain, whereas moderate-to-high rectal doses were associated with late rectal bleeding. These findings may provide practical DVH thresholds that could improve dose optimization strategies in moderate hypofractionated salvage radiotherapy.

## Supplementary Material

Supplementary_materials_rrag045

## Conference presentations

This study was partly presented as a poster presentation at the 38th Annual Meeting of the Japanese Society for Radiation Oncology (JASTRO), Tokyo, Japan, on November 27, 2025.
