# Supplementary material for: DVH parameters and gastrointestinal/genitourinary toxicities in moderate hypofractionated salvage radiotherapy after radical prostatectomy
Source: J Radiat Res. 2026 Jul 7;67(4):656–64. doi: 10.1093/jrr/rrag045 (PMC13400562; doi:10.1093/jrr/rrag045)
Supplement: Supplementary_materials_rrag045 [file supplementary_materials_rrag045.zip › Revised Supplementary data Revision2.docx]

**Supplementary Table S1.** Gastrointestinal and genitourinary toxicity (N = 92).

| Toxicity | | Grade 1 | | Grade 2 | | Grade 3 |  |
| --- | --- | --- | --- | --- | --- | --- | --- |
| **Rectal bleeding** | |  | |  | |  |  |
|  | Acute | 6 (6.5%) | 12 (13.0%) | | 0 (0%) | | |
|  | Late | 22 (23.9%) | 5 (5.4%) | | 8 (8.7%) | | |
| **Anal pain** | |  | |  | |  |  |
|  | Acute | 14 (15.2%) | 22 (23.9%) | | 0 (0%) | | |
|  | Late | 5 (5.4%) | 5 (5.4%) | | 0 (0%) | | |
| **Hematuria** | |  |  | |  | | |
|  | Acute | 0 (0%) | 0 (0%) | | 0 (0%) | | |
|  | Late | 28 (30.4%) | 2 (2.2%) | | 3 (3.3%) | | |

No grade ≥ 4 toxicity was observed.

**Supplementary Table S2.** Univariate and multivariate analyses of toxicities in patients treated without IMRT.

(a) Grade ≥ 2 acute rectal bleeding

|  | Univariate analysis | | | | |  | | | Multivariate analysis | | | |
| --- | --- | --- | --- | --- | --- | --- | --- | --- | --- | --- | --- | --- |
| Factor | *p-*value | | OR | (95% CI) | |  | | *p-*value | | OR | | (95% CI) |
| Anticoagulant use | | 0.91 | 1.14 | | (0.057–7.72) | |  |  | | |  |  |
| HT | | **0.05*** | 4.71 | | (100–33.74) | |  | **<0.001*** | | | 16.08 | (2.28–218.93) |
| DM | | 0.50 | 1.85 | | (0.25–9.31) | |  |  | | |  |  |
| Age | | 0.64 | 1.03 | | (0.90–1.18) | |  |  | | |  |  |
| Hemorrhoid | | 0.93 | 1.08 | | (0.15–5.20) | |  |  | | |  |  |
| ADT | | 0.14 | 4.47 | | (0.56–26.45) | |  |  | | |  |  |
| Pathological stage ≥ T3b | | 0.73 | 0.69 | | (0.04–4.42) | |  |  | | |  |  |
| Anal canal V20Gy (%) | | **0.004*** | 1.04 | | (1.01–1.08) | |  | **0.004*** | | | 1.06 | (1.02–1.12) |

(b) Grade ≥ 2 acute anal pain

|  | Univariate analysis | | |
| --- | --- | --- | --- |
| Factor | *p-*value | OR | (95% CI) |
| Anticoagulant use | 0.35 | 0.40 | (0.02–2.40) |
| HT | 0.85 | 1.11 | (0.38–3.19) |
| DM | 0.96 | 1.04 | (0.21–3.93) |
| Age | 0.47 | 1.04 | (0.94–1.15) |
| Hemorrhoid | 0.29 | 1.88 | (0.57–5.90) |
| ADT | 0.69 | 1.43 | (0.19–7.33) |
| Pathological stage ≥ T3b | 0.45 | 0.56 | (0.08–2.37) |
| Anal canal V40Gy (%) | **<0.001*** | 1.05 | (1.03–1.09) |

| (c) Grade ≥ 2 late rectal bleeding | | | | |  |  |  |  |
| --- | --- | --- | --- | --- | --- | --- | --- | --- |
|  | Univariate analysis | | |  | | Multivariate analysis | | |
| Factor | *p-*value | HR | (95% CI) |  | | *p-*value | HR | (95% CI) |
| Anticoagulant use | **0.01*** | 4.65 | (1.40–15.50) |  | |  |  |  |
| HT | **0.02*** | 4.72 | (1.28–17.44) |  | |  |  |  |
| DM | 0.86 | 1.14 | (0.25–5.22) |  | |  |  |  |
| Age | 0.39 | 1.04 | (0.95–1.15) |  | |  |  |  |
| Hemorrhoid | 0.22 | 0.28 | (0.04–2.17) |  | |  |  |  |
| ADT | 0.90 | 0.88 | (0.11–6.80) |  | |  |  |  |
| Pathological stage ≥ T3b | 0.41 | 1.73 | (0.47–6.38) |  | |  |  |  |
| Grade ≥ 2 acute bleeding | **0.004*** | 5.94 | (1.78–19.77) |  | | **0.005*** | 5.70 | (1.70–19.00) |
| Rectum V60Gy (%) | **<0.001*** | 1.06 | (1.02–1.10) |  | | **0.001*** | 1.06 | (1.02–1.10) |

(d) Grade ≥ 1 late hematuria

|  | Univariate analysis | | |
| --- | --- | --- | --- |
| Factor | *p-*value | HR | (95% CI) |
| Anticoagulant use | 0.28 | 1.71 | (0.65–4.52) |
| HT | 0.79 | 1.11 | (0.52–2.36) |
| DM | 0.50 | 1.36 | (0.55–3.39) |
| Age | 0.49 | 0.98 | (0.92–1.04) |
| ADT | 0.25 | 2.05 | (0.61–6.89) |
| Pathological stage ≥ T3b | 0.20 | 0.39 | (0.09–1.64) |
| Bladder volume | **0.03*** | 1.01 | (1.00–1.01) |

*p* < 0.05 is shown in bold with an asterisk (*).

**Abbreviations:** IMRT = intensity-modulated radiation therapy; OR = odds ratio; CI = confidence interval; Anticoagulant use = anticoagulant and antiplatelet agent use; HT = hypertension; DM = diabetes mellitus; ADT = androgen deprivation therapy; HR = hazard ratio.

**Supplementary Figure S1. Delineation of organs at risk on planning CT.**

The rectum is contoured in brown, the anal canal is contoured in orange, the whole bladder is contoured in yellow, the bladder trigone is contoured in pink, and the bladder neck is contoured in blue.

**Supplementary Figure S2. Biochemical failure**-**free survival (BFFS), overall survival (OS), and metastasis**-**free survival (MFS).**

Kaplan–Meier curves show a 5-year BFFS of 61.8% (a), a 5-year OS of 97.6% (b), and a 5-year MFS of 88.9% (c).

**Supplementary Figure S3. Univariate logistic regression analyses of DVH parameters associated with grade ≥ 1 late hematuria.**

Whole bladder parameters are shown in black, bladder neck parameters in yellow, and bladder trigone parameters in blue. Because the distributions of bladder neck V10Gy and V20Gy were highly skewed, the corresponding odds ratio (OR) estimates were unstable and could not be calculated.
