# Supplementary figures and images for: DVH parameters and gastrointestinal/genitourinary toxicities in moderate hypofractionated salvage radiotherapy after radical prostatectomy
Source: J Radiat Res. 2026 Jul 7;67(4):656–64. doi: 10.1093/jrr/rrag045 (PMC13400562; doi:10.1093/jrr/rrag045)

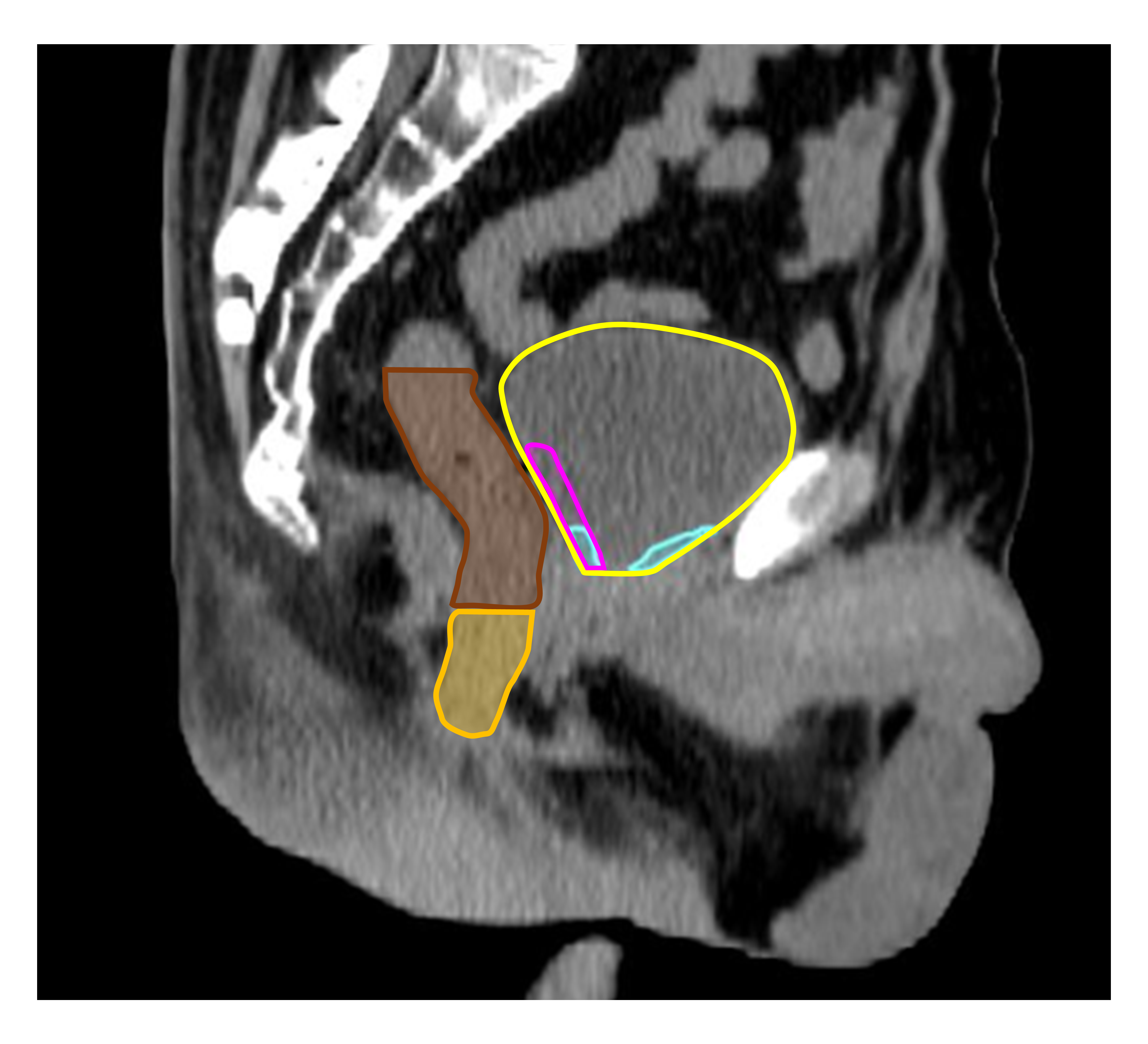

Supplement: Supplementary_materials_rrag045 [file supplementary_materials_rrag045.zip › Revised Supplementary Figure S1.tif]

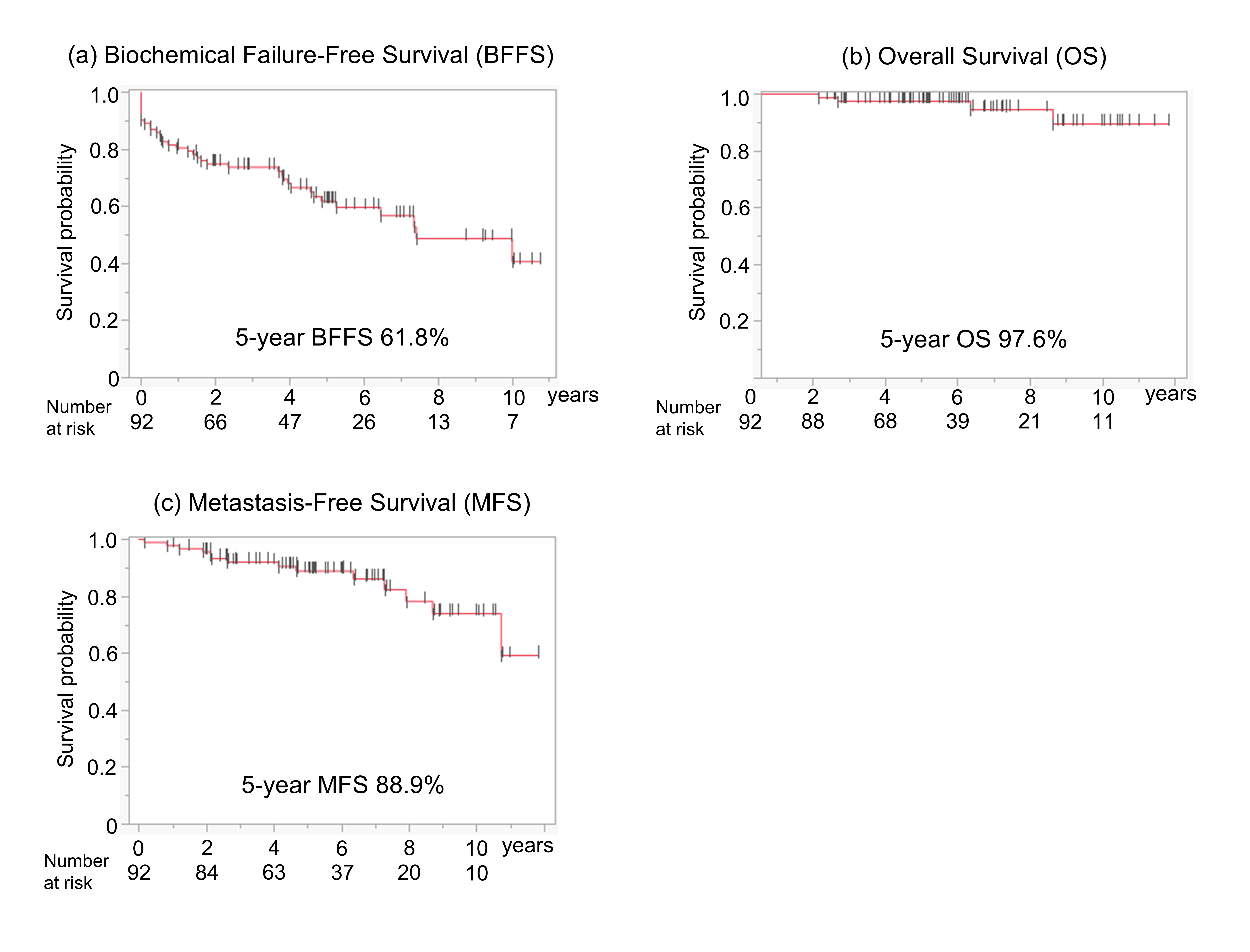

Supplement: Supplementary_materials_rrag045 [file supplementary_materials_rrag045.zip › Revised Supplementary Figure S2.tif]
